# Supplementary material for: Recurrent Vulvovaginal Candidosis and Its Underlying Mechanisms: A Systematic Review
Source: J Fungi (Basel). 2025 May 5;11(5):357. doi: 10.3390/jof11050357 (PMC12112908; doi:10.3390/jof11050357)
Supplement: Supplementary file 1 [file jof-11-00357-s001.zip › jof-3573880-supplementary.pdf]

Journal of *Fungi*

MDPI

Systematic Review

Recurrent Vulvovaginal Candidosis and Its Underlying Mechanisms: A Systematic Review

Maria Lobo [1,†], Catarina Cerqueira [2,†], Acácio Gonçalves Rodrigues [1] and Carmen Lisboa [1,3,*]

[1] RISE-Health, Department of Pathology, Microbiology, Faculty of Medicine, University of Porto, 4200-319 Porto, Portugal; up201906394@med.up.pt (M.L.); agr@med.up.pt (A.G.R.)
[2] Department of Dermatology and Venereology, ULS Braga, 4710-243 Braga, Portugal; catarina.dias.cerqueira@gmail.com
[3] Department of Dermatology and Venereology, ULS São João, 4200-319 Porto, Portugal
* Correspondence: carlis@med.up.pt
† These authors contributed equally to this work.

Supplementary Material S1. Search strategy and study selection criteria

Search strategy: Original search - 1010 results, searched on 21/10/2024

PubMed- 288 results

((((((((((((((((Recurrent[Title/Abstract] OR Persistent[Title/Abstract] OR Chronic[Title/Abstract] OR Recurrence[Title/Abstract] OR Complicated[Title/Abstract]) AND ("vulvovaginal candidiasis"[Title/Abstract] OR "vaginal candidiasis"[Title/Abstract] OR "vulvovaginal candidosis"[Title/Abstract] OR "vaginal candidosis"[Title/Abstract] OR "candida vaginitis"[Title/Abstract] OR "candida vulvovaginitis"[Title/Abstract])) NOT "bacterial vaginosis"[Title/Abstract]) NOT "trichomoniasis"[Title/Abstract]) NOT "trichomonas"[Title/Abstract]) NOT ("quality of life"[Title])) NOT (burden[Title])) NOT (effectiveness[Title])) NOT (efficacy[Title])) NOT (effective[Title])) NOT (safety[Title])) NOT (safe[Title])) NOT (overview[Title])) NOT (description[Title])) NOT ("narrative review"[Title])) NOT ("case report"[Title])) NOT ("editorial"[Publication Type] OR "review"[Publication Type])) AND (("2014"[Date - Publication] : "2024"[Date - Publication]))

Scopus – 386 results<br><br>(TITLE-ABS ("Recurrent" OR "Persistent" OR "Chronic" OR "Recurrence" OR "Complicated") AND TITLE-ABS ("vulvovaginal candidiasis" OR "vaginal candidiasis" OR "vulvovaginal candidosis" OR "vaginal candidosis" OR "candida vaginitis" OR "candida vulvovaginitis") AND NOT TITLE-ABS ("bacterial vaginosis") AND NOT TITLE-ABS ("trichomoniasis") AND NOT TITLE-ABS ("trichomonas") AND NOT TITLE ("quality of life") AND NOT TITLE ("burden") AND NOT TITLE ("effectiveness") AND NOT TITLE ("efficacy") AND NOT TITLE ("effective") AND NOT TITLE ("safety") AND NOT TITLE ("safe") AND NOT TITLE ("overview") AND NOT TITLE ("description") AND NOT TITLE ("narrative review") AND NOT TITLE ("case report") AND NOT DOCTYPE ("ed" OR "re")) AND PUBYEAR > 2013 AND PUBYEAR < 2025
Web of Science- 336 results<br><br>((((((((((((TS=(((((Recurrent OR Persistent OR Chronic OR Recurrence OR Complicated) AND ("vulvovaginal candidiasis" OR "vaginal candidiasis" OR “vulvovaginal candidosis” OR “vaginal candidosis” OR "candida vaginitis" OR "candida vulvovaginitis")) NOT “bacterial vaginosis”) NOT “trichomoniasis”) NOT “trichomonas”)) NOT TI=("quality of life")) NOT TI=(burden)) NOT TI=(effectiveness)) NOT TI=(efficacy)) NOT TI=(effective)) NOT TI=(safety)) NOT TI=(safe)) NOT TI=(overview)) NOT TI=(description)) NOT TI=("narrative review")) NOT TI=("case report")) NOT DT=(Editorial Material OR Review)) AND PY=(2014-2024)
